# Total hip arthroplasty in geriatric patients – a single-center experience

**DOI:** 10.1051/sicotj/2022011

**Published:** 2022-04-04

**Authors:** Philip Mark Anderson, Peter Vollmann, Manuel Weißenberger, Maximilian Rudert

**Affiliations:** University of Wuerzburg, Department of Orthopedics, Orthopädische Klinik König-Ludwig-Haus Brettreichstraße 11 97074 Würzburg Germany

**Keywords:** Total hip arthroplasty, Geriatrics, Health related quality of life, Adverse events

## Abstract

*Background*: As advanced age often leads to accumulating comorbidities, geriatric patients are endangered by serious events during total hip arthroplasty. This study was conducted to explore whether or not the benefit in terms of health-related quality of life (HRQoL) was comparable to younger patients. *Methods*: At a single academic center, 100 patients meeting the following inclusion criteria were retrospectively recruited: (1) primary arthritis of the hip leading to THA; (2) age 80 years or older at the time of surgery; (3) follow up of at least 12 months. For comparison, two further groups were recruited in the same manner, differing only in the age criterion: 100 patients aged 60–69 and 100 patients aged 70–79 at the time of hip replacement. The primary outcome was compared using the WOMAC and the EQ-5D score. The secondary outcome was determined by rates of orthopedic and non-orthopedic complications. Intragroup comparisons of the PROMS were performed by the non-parametric Wilcoxon test for paired samples. For intergroup comparisons of the PROMS, the Kruskal–Wallis-test was performed. Concerning categorial data, intergroup comparisons were performed by the Chi-Square test. The level of significance was set at 0.05. *Results*: Concerning the WOMAC score, neither the absolute values at 12 months after THA (*p* = 0.176) nor the amount of change relative to the values before surgery (*p* = 0.308) differed significantly between the 3 groups. Concerning the EQ-5D index the absolute values at 12 months after THA differed significantly (*p* = 0.008). Rates of orthopedic complications did not differ significantly (*p* = 0.631). Rates of non-orthopedic complications increased significantly with rising age (*p* = 0.033). *Conclusions*: Compared to younger patients, geriatric patients after THA have an equal improvement in hip-specific and general HRQoL. While rates of orthopedic complications are comparable too, non-orthopedic complications occur more frequently.

## Introduction

Total hip arthroplasty (THA) has been named the “operation of the century” [1] in view of the tremendous progress and the achievements made in implanting artificial hip joints during the 20th century. THA is one of the most frequently performed orthopedic operations worldwide due to strong evidence concerning the good results and the benefits for the patients. Nevertheless, orthopedic and non-orthopedic complications still occur in a relevant proportion of patients. Overall, serious complications are rare events, but for certain risk groups, they are known to become more frequent. The risk rises, especially in the presence of multiple comorbidities. As advanced age often leads to accumulating comorbidities, geriatric patients, above all, are endangered by serious events during and after surgery. With this in mind, orthopedic surgeons are frequently confronted how to deal with older patients suffering from severe hip osteoarthritis and who do not respond to conservative treatment. Given the aging population and rising life expectancy worldwide, this problem is anticipated to aggravate [2–4].

The following study was conducted to explore if and to which extent geriatric patients were at higher risk of severe complications during and after THA and whether or not the benefit in terms of health-related quality of life (HRQoL) was comparable to younger patients. According to common definitions, patients aged 80 years or older were defined as geriatric [5].

## Material and methods

Starting backwards with data from 12/31/2018, the first 100 patients meeting the following inclusion criteria were retrospectively recruited: (1) primary arthritis of the hip leading to THA at our institution; (2) age 80 years or older at the time of surgery; (3) follow up of at least 12 months. For comparison, two further groups were recruited in the same manner, differing only in the age criterion: 100 patients aged 60–69 and 100 patients aged 70–79 at the time of hip replacement.

Besides age and gender, the following data were collected:

- Comorbidities.
- Preoperative level of hemoglobin and glomerular filtration rate.
- Technical information about the surgery: type of implant, use of cement, bearing material, surgical approach to the hip.
- ASA score.
- Length of stay at the hospital.
- Follow up.
- Patient-reported outcome measures (WOMAC, EQ-5D) before and 12 months after THA.
- Non-orthopedic complications during hospital stay.
- Orthopedic complications up to the latest follow up.

The primary outcome was compared using prospectively collected patient-reported outcome measures (PROMS) prior to and 12 months after THA. Following the recommendation of “The International Society of Arthroplasty Registries (ISAR) Patient-Reported Outcome Measures (PROMs) Working Group”, one specific and one generic PROM were used, namely the “Western Ontario and McMaster Universities Osteoarthritis Index” (WOMAC) and the “EuroQol 5-dimension” (EQ-5D) score, respectively [6]. The WOMAC has shown especially good measurement properties for arthroplasty patients [7, 8]. Both PROMS were obtained the day before surgery during the preoperative workup. Twelve months after surgery, the questionnaires were sent to the patients’ home addresses.

Concerning the WOMAC, the German version WOMAC-D was used, with the score ranging from 0 as best possible to 240 points as the worst possible outcome. The WOMAC consists of three dimensions: pain (5 items, 0–50 points), stiffness (2 items, 0–20 points), and activity level (17 items, 0–170 points). Each question within the items has a range of 0–10 points on a Likert scale, representing the best to the worst outcome. Minimal clinical important difference (MCID) and patient acceptable symptom state (PASS) have not yet been defined for the used version of the WOMAC. Relying on former investigations showing the MCID to be about 25% of the achievable score [9] and the PASS to be below 1/3 of the maximum score [10], they were defined as follows: 60 points of change for the MCID and a total of 80 points for the PASS, respectively.

Regarding the EQ-5D, the German version of the EQ-5D-3L was used. This generic questionnaire comprises 5 dimensions of health: mobility, self-care, usual activities, pain/discomfort, and anxiety/depression. Each item allows three possible levels of severity to be chosen: no problems, moderate problems, and extreme problems. Through the answers to these questions, 243 different health states can be determined. Population-based preferences for each health state for the German population are available and were used to create an index, with a score of 1 representing perfect health and 0 death [11]. Additionally, the EQ-5D contains a general health visual analog scale (EQ-VAS). Patients mark their general health on the scale, with 0 representing the worst imaginable health state and 100 equaling the best imaginable health state. MCID and PASS of the EQ-5D index after THA have been reported to be 0.31 and 0.92 (0.89 for patients over 70), respectively [12].

The secondary outcome was determined by rates of orthopedic complications up to the latest follow-up and non-orthopedic complications during a hospital stay.

Statistical analysis was performed by SigmaPlot for Windows, version 13.0 (Systat Software Inc., San Jose, CA, USA). Results of the PROMS are presented as the median and 25% and 75% percentile of the data. Intragroup comparisons before and 12 months after surgery were performed by the non-parametric Wilcoxon test for paired samples. For intergroup comparisons of the PROMS, the Kruskal-Wallis-test (ANOVA on ranks) was performed as an omnibus test. In case of significant results, Dunn’s method was used for the following pairwise comparison. Categorial data are presented as rates. Intergroup comparisons of this data were performed by the Chi-Square test. The level of significance was set at 0.05. No correction of significance level was implemented for multiple comparisons in the secondary outcome parameters.

The local ethics committee gave its approval prior to the study (date of approval: 05/25/2020, reference number 2020043001).

## Results

### Study population

The demographics of the study population, the mean length of stay at the hospital, and the mean follow-up are shown in Table 1. The proportions of males being scheduled for THA decreased from 50% in the group 60–69 to 40% in the group ≥ 80.

**Table 1:** Demographics of the study population, length of stay and follow up.

	Age (years) (mean ± SD)	Gender (rate of males)	Length of stay at hospital (days) (mean ± SD)	Follow up (months) (mean ± SD)	Number
Group ≥ 80	82 ± 2	40%	9 ± 3	37 ± 8	100
Group 70–79	75 ± 3	45%	8 ± 4	24 ± 3	100
Group 60–69	65 ± 3	50%	7 ± 2	24 ± 3	100

### Medical conditions

Besides the distribution of the ASA Score, the rate of medical comorbidities, e.g., diabetes mellitus, renal insufficiency, need for anticoagulation, as well as preoperative levels of hemoglobin and the glomerular filtration rate are shown in Table 2. The proportion of patients rated ASA 1, and ASA 2 decreased from 81% in the group 60–69 to 40% in the group ≥ 80.

**Table 2:** Rate of medical comorbidities. Preoperative levels of hemoglobin and glomerular filtration rate.

	Diabetes mellitus (rate)	Renal insufficiency (rate)	Need for anticoagulation (rate)	Preoperative level (g/dL) of hemoglobin (mean ± SD)	Preoperative glomerular filtration rate (mL/min) (mean ± SD)	ASA Score (rates)
Group ≥ 80	15%	9%	25%	13.5 ± 1.4	73.4 ± 20.5	ASA 1: 1%
						ASA 2: 39%
						ASA 3: 58%
						ASA 4: 1%
						Not recorded: 1%
Group 70–79	16%	8%	18%	14.0 ± 1.4	76.0 ± 18.1	ASA 1: 1%
						ASA 2: 55%
						ASA 3: 41%
						ASA 4: 1%
						Not recorded: 2%
Group 60–69	12%	3%	8%	14.5 ± 1.2	84.8 ± 18.8	ASA 1: 5%
						ASA 2: 76%
						ASA 3: 19%
						ASA 4: 0%

### Technical aspects

All THAs were performed via the direct anterior approach to the hip in the supine position. In all cases of cementless implantation, “Allofit-S Alloclassic” cups (ZimmerBiomet) with “Durasul alpha” liners and “M/L Taper” stems (ZimmerBiomet) were used. In cases of cementation, the “Chirulen” cup (Aesculap) and the “M.E. Müller Geradschaft” (ZimmerBiomet) were used. The decision concerning the bearing material of the head (ceramic vs. metal) was made by the responsible surgeon after consultation with the patient. Table 3 provides an overview of the rates of different fixation techniques and bearing materials depending on age.

**Table 3:** Fixation technique and bearing material of the femoral head.

	Fixation technique (rates)			Bearing material of the head (rates)	
	Cementless	Hybrid	Cemented	Ceramic	Metal
Group ≥ 80	61%	35%	4%	64%	36%
Group 70–79	93%	7%	0%	96%	4%
Group 60–69	98%	0%	2%	99%	1%

Age and the type of fixation (cementless vs. hybrid and fully cemented) were significantly related (*p* < 0.001). Additionally, age and the bearing type (metal vs. ceramic) were significantly related too (*p* < 0.001).

### Outcome

Within the 3 groups, the WOMAC Score and each of its items showed significant improvements 12 months after surgery compared to the state before THA (Tables 4–6).

**Table 4:** WOMAC score and items before and after THA in the group ≥ 80.

Group ≥ 80	Before THA Median (25–75%)	12 months after THA Median (25–75%)	Absolute difference Median (25–75%)	Relative difference to baseline Median (25–75%)	*p-*value
WOMAC					
Response rate: 74%					
Pain (0–50)	27.0 (21.5–35.3)	4.0 (0.0–12.0)	18.0 (12.0–28.0)	84.0% (55.2–100%)	<0.001
Stiffness (0–20)	11.5 (7.8–15.0)	4.0 (0–6.5)	7.0 (2.0–12.0)	63.6% (36.4–100%)	<0.001
Physical function (0–170)	102.0 (72.3–130.1)	30.6 (7.2–54.4)	61.2 (34.0–95.2)	67.6% (41.8–93.7%)	<0.001
Score (0–240)	140.9 101.6–176.9)	39.4 (8.9–66.4)	79.0 (55.8–136.7)	67.4% (43.4–94.9%)	<0.001

**Table 5:** WOMAC score and items before and after THA in the group 70–79.

Group 70–79	Before THA Median (25–75%)	12 months after THA Median (25–75%)	Absolute difference Median (25–75%)	Relative difference to baseline Median (25–75%)	*p*-value
WOMAC					
Response rate: 81%					
Pain (0–50)	26.0 (20.0–34.0)	2.0 (0–9.5)	21.0 (12.0–27.3)	90.0% (64.8–100%)	<0.001
Stiffness (0–20)	11.0 (8.0–14.0)	3.0 (0.0–6.0)	6.0 (3.0–10.0)	66.7% (44.8–100%)	<0.001
Physical function (0–170)	94.4 (69.7–120.7)	18.7 (3.4–39.1)	64.6 (45.5–93.5)	76.8% (57.9–96.0)	<0.001
Score (0–240)	130.9 (98.5–169.9)	25.5 (5.1–54.9)	95.7 (60.3–134.2)	76.5% (61.2–95.2)	<0.001

**Table 6:** WOMAC score and items before and after THA in the group 60–69.

Group 60–69	Before THA Median (25–75%)	12 months after THA Median (25–75%)	Absolute difference Median (25–75%)	Relative difference to baseline Median (25–75%)	*p*-value
WOMAC					
Response rate: 76%					
Pain (0–50)	24.5 (18.0–33.0)	2.0 (0.0–10.0)	18.0 (10.1–24.0)	92.0% (56.6–100%)	<0.001
Stiffness (0–20)	12.0 (7.0–15.0)	3.5 (0.0–6.0)	8.0 (4.0–11.0)	69.2% (45.3–100%)	<0.001
Physical function (0–170)	90.1 (62.1–110.5)	13.6 (0.4–39.1)	56.1 (39.1–79.9)	82.6% (54.1–100%)	<0.001
Score (0–240)	126.0 (88.1–155.0)	18.4 (1.7–56.6)	83.8 (62.8–105.8)	81.0% (57.2–99.0%)	<0.001

**Figure 1:**
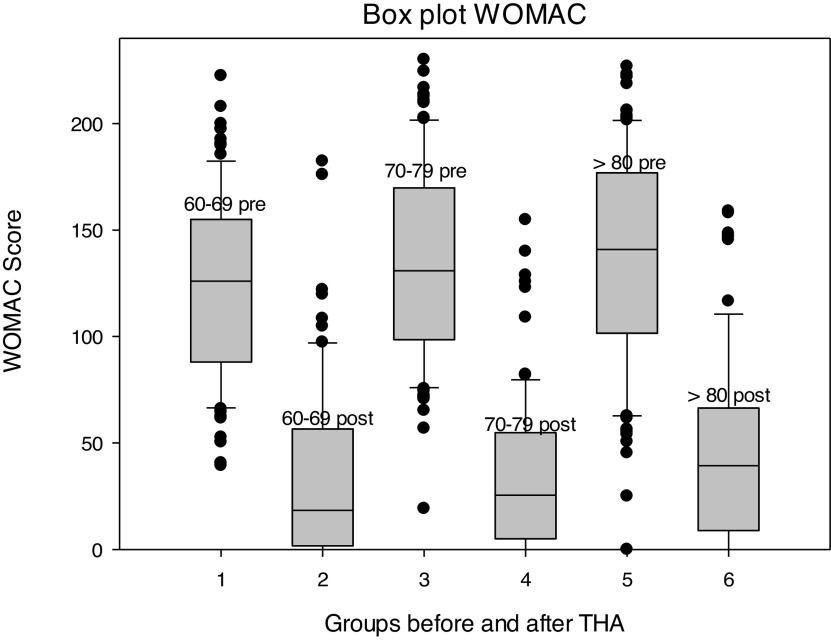
Box plot WOMAC.

Concerning the WOMAC score, neither the absolute values at 12 months after THA (*p* = 0.176) nor the amount of change relative to the values before surgery (*p* = 0.308) differed significantly between the 3 groups (Figure 1). Relative to the baseline, the biggest improvement was achieved within the subscale “pain” followed by “physical function” in all 3 groups. The total score showed improvement of at least 2/3 compared to the state before THA in all 3 groups. This amount of change was clinically meaningful in at least 75% of patients, and values within the PASS were achieved in the majority of patients (Table 7).

**Table 7:** Results of the WOMAC score compared to PASS and MCID.

	Difference exceeding the MCID (%)	Postoperative values within the PASS (%)
Group **≥** 80	74	76
Group 70–79	74	89
Group 60–69	79	86

The EQ-5D index score and EQ-VAS too improved significantly (Tables 8–10) in all three groups. Response rates were above 70% in all groups.

**Table 8:** EQ-5D index score and VAS before and after THA in the group ≥ 80.

Group ≥ 80	Before THA Median (25–75%)	12 months after THA Median (25–75%)	Absolute difference Median (25–75%)	Relative difference to baseline Median (25–75%)	*p*-value
EQ-5D					
Response rate: 71%					
Index (0–1)	0.495 (0.291–0.613)	0.770 (0.606–1.000)	0.262 (0.152–0.446)	65.0% (23.6–136.2%)	<0.001
VAS (0–100)	50.0 (30.0–50.0)	80.0 (50.0–90.0)	30.0 (10.0–48.8)	60.0% (14.9–100.0%)	<0.001

**Table 9:** EQ-5D index score and VAS before and after THA in the group 70–79.

Group 70–79	Before THA Median (25–75%)	12 months after THA Median (25–75%)	Absolute difference Median (25–75%)	Relative difference to baseline Median (25–75%)	*p*-value
EQ-5D					
Response rate: 76%					
Index (0–1)	0.495 (0.291–0.606)	0.902 (0.747–1.000)	0.394 (0.250–0.492)	65.0% (47.1–207.6%)	<0.001
VAS (0–100)	50.0 (30.0–60.0)	80.0 (70.0–90.0)	40.0 (20.0–52.5)	80.0% (28.6–183.3%)	<0.001

**Table 10:** EQ-5D index score and VAS before and after THA in the group 60–69.

Group 60–69	Before THA Median (25–75%)	12 months after THA Median (25–75%)	Absolute difference Median (25–75%)	Relative difference to baseline Median (25–75%)	*p*-value
EQ-5D					
Response rate: 71%					
Index (0–1)	0.606 (0.291–0.613)	1.000 (0.750–1.000)	0.296 (0.231–0.397)	50.2% (32.8–102.0%)	<0.001
VAS (0–100)	50.0 (30.0–60.0)	80.0 (70.0–100)	30.0 (18.8–50.0)	60.0% (25.0–133.3%)	<0.001

**Figure 2:**
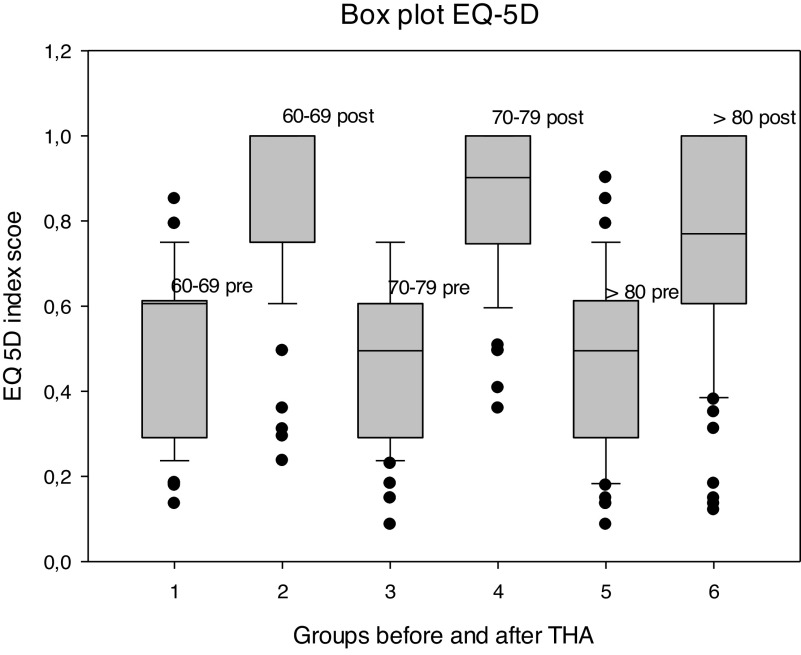
Box plot EQ-5D.

Concerning the EQ-5D index, the absolute values at 12 months after THA differed significantly in the omnibus test (*p* = 0.008). Following pairwise comparisons showed significantly lower values in the group ≥ 80 compared to the group 60–69 (*p* < 0.05). In contrast, the amount of change between the values before and 12 months after surgery did not differ significantly between the 3 groups (*p* = 0.083) (Figure 2). The same results could be observed with the EQ-VAS: significant differences in the absolute values after 12 months (*p* = 0.017) due to lower values of the group ≥ 80 compared to the group 60–69 (*p* < 0.05). But no significant difference in the amount of change relative to the values before THA (*p* = 0.158).

When using the above-mentioned thresholds for MCID and PASS, only around half of the patients experienced clinically important differences of the EQ-5D index leading to an acceptable symptom state in only 44–60%, depending on age (Table 11).

**Table 11:** Results of the EQ-5D index compared to PASS and MCID.

	Difference exceeding the MCID (%)	Postoperative values within the PASS (%)
Group ≥ 80	44	44
Group 70–79	60	60
Group 60–69	49	52

Rates of orthopedic complications were low in all 3 groups and did not differ significantly (*p* = 0.631; Table 12). The most common orthopedic complication was dislocation in the groups 60–69 (2%) and 70–79 (3%), whereas periprosthetic fracture was the most frequent orthopedic complication in the group ≥ 80 (2%). In contrast, rates of non-orthopedic complications (internal, neurological, psychiatric, urological, and others) increased significantly with rising age (*p* = 0.033; Table 12). Rates of blood transfusion, too, were significantly related to advancing age (*p* = 0.032). Overall, one patient died 2 days after THA because of mesenteric ischemia.

**Table 12:** Rates of orthopedic complications, non-orthopedic complications, blood transfusion, and mortality.

	Orthopedic complications (rate)	Non-orthopedic complications (rate)	Blood transfusion (rate)	Mortality (rate)
Group ≥ 80	8%	13%	11%	0%
Group 70–79	8%	8%	6%	1%
Group 60–69	5%	3%	2%	0%

## Discussion

The data presented in this study show that geriatric patients aged 80 years and older improve just as much as younger patients in terms of HRQoL by THA. This was not only true for hip-specific HRQoL measured by the WOMAC score but also for generic HRQoL as assessed by the EQ-5D score. While improving the general quality of life equally in the geriatric patients, THA did not achieve equal absolute values in the EQ-5D 12 months after surgery compared to patients under 70 years old. This is not surprising considering the higher burden of comorbidities with increasing age, as can be seen, for example, in the rate of 25% of geriatric patients needing anticoagulation in contrast to 8% in the group under 70 years (Table 2). THA, of course, cannot solve all the problems caused by age-related comorbidities and can therefore not ensure an identical quality of life compared to younger patients. Additionally, the higher rate of non-orthopedic complications within the group aged 80 years and older might have affected the general life quality even 12 months after surgery.

Aalund et al. [13] published an extensive series of 1283 THA in 2017 showing a positive correlation between age and an increase of HRQoL at 12 months after THA as measured by the EQ-5D-3L in a Danish population. Although not discussed in their paper, the absolutes values of the EQ-5D index in their study were lower in the patients over 79 than in the younger patients, therefore confirming our results. The authors concluded that age should not be of concern when estimating the potential benefits of HRQoL when preoperatively making a decision. Nevertheless, complications during and after THA were not assessed. Similarly, Austin et al. [14] recently demonstrated equal functional outcomes of patients older than 80 years undergoing THA compared to younger patients measured by the Patient-Reported Outcome Measurement Information System (PROMIS)-10 physical component summary score. Again, rates of complications were not reported. Furthermore, the authors found the patients aged 80 years and older to stay longer at the hospital after surgery and therefore use more resources as the younger patients. This is confirmed by our results showing the patients > 80 to stay in average 2 days longer at the hospital than the patients aged 60–69 (Table 1). At the moment, there is no financial compensation for this in the German DRG system.

When interpreting PROMS, numeric changes of the scores must always be correlated to their clinical meaningfulness, for example, by using constructs like the PASS and the MCID. In this study, compared to their younger counterparts, geriatric patients were more likely not to achieve the PASS in both the WOMAC and the EQ-5D. Rates of patients reaching the PASS of the EQ-5D were remarkably low in all three groups. This was even more surprising when considering the median of relative change to baseline exceeding 50% in all groups. When interpreting these findings, several limitations must be taken into account: first, the PASS is known to be lower for older patients, maybe corresponding to their lower level of activity and expectations [12]. Second, MCID and PASS were adopted from studies not investigating the German population. Transferring the results between different populations might not be adequate. Third, PASS and MCID for the WOMAC were adopted from studies using other scaling systems (0–100) than the one used in this study (0–240). This might lead to misinterpretation of the results. Fourth, improvements in general health after THA are known to be lower than hip-specific improvements [9]. And finally, a recent systematic review showed high variability of reported thresholds for PASS and MCID of the WOMAC, therefore mitigating the reliability of these values [15].

In contrast to the comparable benefit in terms of improvement of HRQoL, the geriatric patients in this study were at higher risk of peri- and postoperative adverse events. While rates of orthopedic complications did not differ significantly, internal, urological, psychiatric and other complications were more than four times more frequent in the geriatric patients compared to the patients under 70. Similarly, rates of blood transfusion rose by up to 11% in the group over 80 years, while being rare in patients aged less than 70 years. This was evident, although mean preoperative levels of hemoglobin were similar in all groups. Yohe et al. recently reported elevated rates of transfusion for octogenarians undergoing THA, too [16]. Besides lower tolerance of anemia in the elderly, the indication for transfusion could have been made more frequently by the responsible physician since, for example, chronic HIV and hepatitis - possibly induced by transfusion – may not be of that much concern in geriatric patients. Nevertheless, the literature does not support this approach as clear evidence of benefits is lacking [17].

Our findings confirm former studies showing an elevated vulnerability of geriatric patients undergoing THA to non-orthopedic complications, especially when age is related to a higher burden of comorbidities [3, 18–20]. Age over 80 has even been shown to be an independent risk factor for mortality and complications after THA [20]. Therefore, in a similar manner as geriatric involvement in trauma surgery has been intensified recently [21], involving geriatricians in the preoperative workup and the postoperative care might be beneficial in this special patient group undergoing THA [22]. Further research is required to investigate if major medical complications can be reduced by so-called orthogeriatric care.

Besides the results in terms of HRQoL and complication rates, the existing data prove age-adapted modifications of the technical procedure of THA among orthopedic surgeons. First, the use of cement, especially at the stem, increased significantly by age (Table 3). This finding reveals an awareness of the higher rates of intraoperative and early postoperative periprosthetic fractures reported previously by the use of cementless stems in older patients [23, 24]. Consistent with this, both cases of early periprosthetic fractures in the octogenarians in this study occurred after implanting cementless stems. Second, the use of metalheads against highly cross-linked polyethylene instead of ceramic heads increased significantly with age (Table 3). While the literature shows similar mid-to-long-term results of the two bearing types [25], the costs of metalheads is lower. This finding indicates that orthopedic surgeons might be susceptible to economic issues during surgery in cases where data that give clear recommendations on how to decide are absent.

When interpreting the results of this study, one must be aware of the limitations mostly due to the retrospective design. Selection bias – excluding highly comorbid patients – when indicating surgery in the older patients might have occurred. Furthermore, the choice of fixation type and bearing material was made by the responsible surgeon without documented decision criteria. Therefore, the underlying considerations must be interpreted cautiously. As usual in retrospective studies, including patient reports, the response rate concerning the PROMS 12 months after surgery was lower than 100%. Reporting bias, therefore, cannot be ruled out. Finally, no statistical correction of significance level was implemented for multiple comparisons in the secondary outcome parameters, possibly accepting a higher risk of false-positive findings.

On the other hand, this study work has several strengths. First, the primary outcome was examined by two widely used PROMS, ensuring a broad understanding of the general and hip-specific quality of life of the participants of this study. In contrast, many former studies on the results of THA in geriatric patients solely relied on rates of complications and radiographic examinations, therefore excluding the patients’ perspectives on this procedure. Furthermore, the PROMS were collected in a prospective manner, reducing the problems that are inherent to retrospective studies. Second, this study includes a large number of patients at a single high-volume university institution who were operated on in a highly standardized manner, including the type of implants. Confounders by differing surgical approaches, materials, and skills are therefore unlikely to have occurred.

## Conclusion

Compared to younger patients, geriatric patients > 80 after THA have an equal improvement in hip-specific and general HRQoL. While rates of orthopedic complications are comparable too, non-orthopedic complications occur more frequently. Further study work is necessary to investigate the possible benefits of orthogeriatric care in this field.

## Conflict of interest

The authors declare that they have no relevant financial or non-financial interests to report.

## Funding

This publication was supported by the Open Access Publication Fund of the University of Wuerzburg.

## Ethical approval

The local ethics committee of the University of Wuerzburg gave its approval prior to the study (date of approval: 05/25/2020, reference number 2020043001).

## Informed consent

This article does not contain any studies involving human subjects.

## Authors’ contributions

PA set up this study. PV collected the data. PA and PV were responsible for analyzing and interpreting the data. PA wrote this manuscript. MR and MW were responsible for revising and modifying the manuscript.
